# Implementation of non-pharmacological interventions for the treatment of hypertension in primary care: a narrative review of effectiveness, cost-effectiveness, barriers, and facilitators

**DOI:** 10.1186/s12875-022-01884-8

**Published:** 2022-11-24

**Authors:** Raja Ram Dhungana, Zeljko Pedisic, Maximilian de Courten

**Affiliations:** 1 Institute for Health and Sport, Victoria University, Melbourne, Australia; 2 Melbourne School of Population and Global Health, University of Melbourne, Melbourne, Australia

**Keywords:** Hypertension, Primary care, Alcohol reduction, Sodium reduction, Potassium supplementation, Physical activity, Weight reduction, Healthy diets, Non-pharmacological intervention

## Abstract

**Background:**

The current guidelines for the prevention, detection, evaluation, and management of hypertension recommend six types of non-pharmacological interventions: alcohol reduction, salt intake reduction, increased potassium intake, physical activity, weight loss, and heart-healthy diets. However, the non-pharmacological interventions are still not widely used in primary care. In this paper, we, therefore, reviewed and summarised the evidence on the effectiveness, cost-effectiveness, barriers, and facilitators of non-pharmacological interventions for the treatment of hypertension in primary care.

**Methods:**

A thorough literature search was conducted in Embase, Google Scholar, and PubMed databases, to identify the most recent reviews or, in their absence, primary studies on alcohol reduction, salt intake reduction, potassium supplementation, physical activity, weight reduction, heart-healthy diets, and other non-pharmacological interventions for the treatment of hypertension in primary care.

**Results:**

Alcohol reduction is a non-pharmacological intervention for the treatment of hypertension in primary care with proven effectiveness, feasibility, and acceptability. Interventions for sodium intake reduction, physical activity, and weight reduction are effective but there is insufficient evidence regarding their feasibility and acceptability in primary care settings. Evidence on the effectiveness of potassium intake and heart-healthy diets is limited and inconsistent. There is a lack of evidence on the cost-effectiveness of non-pharmacological interventions in the treatment of hypertension. The most common barriers to deliver such interventions related to healthcare providers include a lack of time, knowledge, self-confidence, resources, clear guidelines, and financial incentives. The most common barriers related to patients include a lack of motivation and educational resources. Less evidence is available on facilitators of implementing non-pharmacological interventions in primary care. Besides, facilitators differed by different types of interventions.

**Conclusions:**

Available evidence suggests that more pragmatic, clinically feasible, and logistically simple interventions are required for sodium intake reduction, physical activity, and weight reduction in primary care settings. Future studies should provide further evidence on the effectiveness of weight control, potassium intake, and heart-healthy diets. More research is also needed on cost-effectiveness and facilitators of all types of effective non-pharmacological interventions for the treatment of hypertension in primary care.

## Background

There is a wealth of literature on alcohol intake, high salt intake, low potassium intake, physical inactivity, obesity, and unhealthy diet as key determinants of high blood pressure. A recent systematic review demonstrated that consumption of five or more standard alcoholic drinks per day is associated with a 74% greater risk for hypertension [1]. A pooled analysis of 133,118 individuals showed that an additional gram of dietary sodium intake per day is associated with an average increase in systolic blood pressure of 2.58 mmHg [2]. Low potassium intake was found to be associated with an increased risk of hypertension [3]. Around 6% of hypertension cases can be attributed to low potassium intake [3]. Likewise, physical inactivity was found to be associated with an increased risk of hypertension [4]. The population attributable fraction of hypertension due to physical inactivity is 13% [4]. Jayadi et al. [5] performed a meta-regression analysis with a pooled sample of 2.3 million participants from 57 prospective cohort studies and found a significant positive relationship between body mass index (BMI) and blood pressure. A five-unit increase in BMI was associated with a 49% higher risk of hypertension [5]. Furthermore, high intake of red meat, processed meat, and high-fat dairy products and low intake of fruit and vegetables may increase the risk of hypertension [6, 7]. For example, red meat consumption is associated with 22% increased risk of hypertension [8]. Importantly, these risk factors are modifiable, and their modification (i.e. reducing alcohol consumption, reducing salt intake, increasing potassium intake, increasing physical activity, reducing body weight, and improving diet) may, therefore, play an important role in the prevention and management of high blood pressure.

A recent review found that a reduction in alcohol of 16–80% across 14 intervention trials was associated with an average reduction in systolic blood pressure of 3 mmHg [9]. The effect seems to be greater in hypertensive and medicated patients [9], and among those who drink more than two standard alcoholic drinks per day [10]. Several systematic reviews have consistently reported that salt reduction is associated with a significant reduction in blood pressure [11–18]. Salt reduction strategies were found to be associated with an average change in systolic blood pressure of -3.9 mmHg to -5.9 mmHg [11–18]. The blood pressure-lowering effect induced by a reduction in salt intake was found to be greater among hypertensive individuals [17]. The negative effect of high salt intake on blood pressure was found to be attenuated by potassium supplementation, as it may facilitate the removal of excess sodium from the body. The importance of potassium supplementation for blood pressure reduction has been substantiated by the findings of a systematic review [19]. The study reported that an increased potassium intake achieved through changes in diet or the use of dietary supplements was associated with an average reduction in systolic blood pressure of 4.7 mmHg, and that the reduction was greater among those who consumed more dietary salt [19]. Likewise, various types of physical activity, such as aerobic exercise [20], isometric and dynamic resistance training [21–24], and light-intensity incidental physical activity such as standing or walking at work [25, 26] are significantly associated with blood pressure reduction. Interventions that caused any weight loss were found to be associated with an average reduction in systolic blood pressure of 2.68 mmHg [27]. Furthermore, heart-healthy diets, such as Dietary Approaches to Stop Hypertension (DASH) diet, Mediterranean diet, low-carbohydrate diet, diet with low-glycaemic index, low-sodium diet, and low-fat diet were found to be effective in reducing blood pressure in hypertensive and pre-hypertensive individuals [28].

Taking into account the evidence on their effectiveness, the current guidelines for the prevention, detection, evaluation, and management of hypertension issued by the American College of Cardiology and American Heart Association recommend six types of non-pharmacological interventions, including alcohol intake reduction, salt intake reduction, increased potassium intake, physical activity, weight loss, and heart-healthy diets [29]. The International Society of Hypertension guidelines also highlighted the importance of non-pharmacological interventions and recommended them to be used along with the antihypertensive medications for optimum control of hypertension [30]. Additionally, growing evidence suggests that some of the non-pharmacological interventions could help reduce the needed dosage of antihypertensive medication or result in a greater reduction in blood pressure if they are used combined with medications [31–33]. The body of evidence on other non-pharmacological interventions, such as yoga, healthy drinks, and stress reduction, is also growing [30, 34].

However, the non-pharmacological interventions are still not widely used in primary care. For example, less than one in four general practitioners in France, Germany, Italy, Spain, and the UK assesses alcohol intake and recommend alcohol reduction among their hypertensive patients [35]. Similarly, around one-third of primary care providers in the USA reported that their patients were unlikely to comply with the advice to reduce salt intake [36]. Also, in the UK, 90% of the overweight patients did not receive weight management intervention at primary care [37].

In this paper, we, therefore, thoroughly reviewed and summarised the evidence on the effectiveness, cost-effectiveness, barriers, and facilitators of non-pharmacological interventions for the treatment of hypertension in primary care and provided recommendations for future research in this area.

## Methods

A thorough literature search was conducted in Embase, Google Scholar, and PubMed databases. The search keywords included “hypertension” or “blood pressure” combined with keywords for common non-pharmacological interventions for hypertension including “non-pharmacological intervention”, “lifestyle modification”, “alcohol”, “salt”, “potassium”, “physical activity”, “weight”, “diet”, and “DASH”. Forward and backward reference searches were performed to identify additional relevant studies.

The most recent review papers or, in their absence, primary studies on alcohol reduction, salt reduction, potassium intake, physical activity, weight control, and heart-healthy diets in primary care were included in the review.

Key findings from the included papers in regard to non-pharmacological intervention for the treatment of hypertension in primary care were extracted. We focused on the effectiveness and cost-effectiveness of the interventions and on barriers to and facilitators to their implementation in primary care. When extracting findings on barriers and facilitators, we relied on the categorisations that were originally provided in the included studies. The extracted data were narratively summarised.

## Alcohol reduction

Brief alcohol interventions with the aim to reduce alcohol consumption have shown to be effective when delivered in the primary care setting [38]. A systematic review found that this intervention reduces alcohol intake by on average 38 g per week [39]. Kaner et al. [40], in their recent systematic review, found a slightly lower overall effect size. The participants who received a brief intervention reduced the alcohol intake on average by 26 g. The intervention was found to be more effective among the individuals who are at a lower risk of alcohol dependence [41, 42], or if the intervention is delivered by a nurse [43]. A recent study also suggested that hypertensive patients at primary care could benefit from a brief alcohol intervention delivered by physicians with the aim to reduce blood pressure [41]. Rehm et al. recommended several strategies to reduce alcohol intake among hypertensive patients in primary care [44]. The recommendations include screening for harmful alcohol use and applying Brief Advice [alcohol reduction] for newly diagnosed or untreated hypertensive patients in primary care [44]. Studies have shown that implementing a brief alcohol intervention in the primary healthcare setting is a cost-effective strategy to reduce alcohol consumption [45]. However, evidence on the effectiveness of this intervention among individuals with severe alcohol dependence, women, older adults, younger adults, minority groups, and those from low- and middle-income countries is scarce [38, 42].

Several challenges have been identified when implementing brief alcohol interventions in the primary care setting (Table 1). The common barriers to its implementation include physician’s time constraints, hesitancy to provide counselling to patients on alcohol reduction, the stigma attached to alcohol use, and a lack of skills and knowledge [46–48]. The use of electronic devices and mobile phones to deliver the intervention may address some of the barriers in the implementation process [49–52], but further research is required to confirm their usefulness specifically in the primary care setting. Likewise, it was suggested that specialised training, support, and financial reimbursement could also encourage medical practitioners in primary care units to routinely assess patients’ alcohol consumption [48, 53]. Furthermore, delegating work to a non-physician specialist and tailoring interventions to patient needs could also facilitate the implementation of brief alcohol interventions in primary care [48].

**Table 1:** Summary of findings on non-pharmacological interventions for the treatment of hypertension in primary care

Type of intervention	Common implementation strategies in primary care	Key findings on effectiveness, barriers, and facilitators
Alcohol intake reduction	1. Brief alcohol intervention	Effectiveness Average reduction in alcohol intake: 26 g/week (95% CI: ‐37, ‐14) [40] Rose et al. [54] found a reduction of 4.2 mmHg in systolic blood pressure and 3.3 mmHg in diastolic blood pressure among hypertensive patients who received a brief alcohol intervention Cost-effectiveness The incremental cost-effectiveness ratio of implementing a brief alcohol intervention for alcohol reduction in primary care was found to be at least AU$650 per QALY/life-year gain [45] Barriers Existing workload, limited resources and support, and perceived lack of knowledge and confidence among providers [48] Facilitators Adequate resources, availability of training for providers, and tailoring interventions to patient needs [48]
Salt intake reduction	1. Dietary counselling	Effectiveness Dietary counselling led to average reduction in sodium intake of 73 to 93 mmol/day across the intervention groups vs. 3.2 to 12.5 mmol/day among controls. An average reduction in blood pressure was -4 to -27 mmHg [55] Cost-effectiveness No published evidence found Barriers Poor adherence to low-salt diet among patients [56], low self-efficacy among patients [57], difficulties associated with monitoring salt intake in primary care [58], perceived lack of time among primary care workers, and lack of reimbursement for providing the service [36] Facilitators No published evidence found
Potassium intake	1. Advice to increase intake of potassium-rich fruit and vegetables 2. Advice to use potassium-containing supplements	Effectiveness Findings on the effect of advice-based interventions promoting potassium rich fruit and vegetables intake on blood pressure in primary care settings are inconsistent [59–61] No published evidence was found about the effect of potassium supplementation on blood pressure in primary care settings. However, a systematic review found that potassium supplementation decreases systolic blood pressure on average by 4.48 mmHg in hypertension [62] The study did not specify the study setting(s) Cost-effectiveness No published evidence found Barriers Low patient motivation, lack of provider time, and lack of educational resources for patients Facilitators No published evidence found
Physical activity	1. Brief Intervention 2. Exercise referral schemes	Effectiveness Brief intervention resulted in a small increase in physical activity (standardized mean difference of 0.17) [63] Exercise referral schemes resulted in an increase in physical activity of on average 55 min per week [64] Cost-effectiveness The incremental cost per QALY of Brief Intervention is AU$ 3160 [65] Exercise referral led to an increase of 0.003 quality-adjusted life-years (QALYs) at an additional cost of AU$ 458 per person, typically per 10–12 weeks of intervention [64] Barriers Lack of time, limited resources, and lack of financial incentives for healthcare workers [63, 66] Lack of professional guidance when learning how to exercise and while exercising, lack of peer support, lack of family and social support, and lack of motivation for patients [63, 66, 67] Facilitators Health workers’ perception of physical activity as an effective intervention, and financial incentives for healthcare workers [63, 66, 67]
Weight reduction	1. Behavioural therapy 2. Restrictive diet	Effectiveness Behavioural therapy led to an average weight reduction of 1.4 kg [68] Compared with a behavioural programme alone, very low energy diet combined with a behavioural programme reduced weight by 3.9 kg at one year, 1.4 kg at two years, and 1.3 kg at 38–60 months [69] Cost-effectiveness No published study was found on cost-effectivenss analysis. However, a study reported the cost of behavioural therapy as AU$ 170 per one kilogram of weight lost [70] The incremental cost-effectiveness ratio of low energy dietary replacement was AU$ 5882 (4738–7060), assuming that the weight reduced by one kilo is maintained for at least 5 years [71] Barriers Lack of self-motivation, lack of self-control, inability to afford healthy foods and exercise equipment, inability to resist the temptation to eat ‘junk’ food, competing priorities, and comorbidities in patients [72, 73]. Reluctance to discuss weight management with patients, insufficient confidence, knowledge and skill to help patients manage their weight, lack of clear guidelines for weight management in primary care, and limited resources and time among health professionals [73, 74] Facilitators Peer support, professional support, social support, self-motivation to adhere to the dietary intervention, incentives and rewards are facilitators found for patients [73, 74]
Heart-healthy diets	Dietary counselling	Effectiveness Inconsistent findings on the effectiveness of diets for blood pressure reduction in primary care settings Cost effectiveness No published evidence found Barriers Low patient motivation, lack of provider time, lack of educational resources for patients [75], difficulty in assessing patient’s dietary pattern, patient’s non-adherence to dietary advice, inconsistent dietary guidelines [76] Facilitators Facilitators for physician-delivered dietary advice for patients with hypertension are using electronic medical record tools that support dietary screening or counselling, access to dietitian support, and availability of educational resources for physicians

## Salt intake reduction

Informational interventions and dietary counselling are the most common strategies applied to reduce salt intake in hypertensive patients [77].

Hooper et al. [78], in their systematic review, demonstrated that advice-based intervention for salt reduction was significantly associated with a reduction in systolic blood pressure of on average 1.1 mmHg and urinary sodium excretion of on average 35.5 mmol/day at 13- to 60-month follow-ups. Similarly, Ferrara et al. [79] investigated the effectiveness of a lifestyle education intervention among 188 hypertensive patients at an outpatient clinic in Italy over a period of two years. They found that the intervention significantly reduced sodium intake and systolic blood pressure [79]. Lin et al. [80] also assessed the effects of a physician and patient targeted education intervention for reducing blood pressure among hypertensive patients in a clinical setting. Both patient and physician targeted interventions significantly reduced sodium intake and blood pressure [80].

In a systematic review, Ruzicka et al. [55] evaluated the feasibility of implementing effective sodium reduction strategies to treat hypertension in the primary care setting. The interventions that were not limited to mere counselling, but included provision of food, prepared meals, or intensive inpatient training sessions were difficult to be implemented by primary care providers due to a lack of time.

Alternatively, clinically feasible and logistically simple method such as single-session dietary counselling by dieticians in the outpatients setting could be effective for reducing salt intake [81]. However, further studies are required to test the effectiveness and cost effectiveness of more structured outpatient dietary counselling methods for salt reduction in the primary care setting.

Low adherence to sodium reduction interventions is a key barrier for their implementation in primary care [56]. The low adherence of patients to such interventions is usually due to their poor knowledge, attitude, and behaviour related to dietary salt intake [56, 82]. Some of the reasons for non-adherence to dietary advice are a lack of clear labelling of food products and limited choice of low-salt foods [83] and low self-efficacy for low sodium diet among hypertensive individuals [57]. A systematic review found that people are not fully aware that the food they are eating daily, such as bread and rolls, pizzas, sandwiches, tacos and burritos, cured meats and cold cuts, chicken, eggs and omelettes, soups, and cheese often contain a high amount of salt [82, 84]. Some studies have shown that even those who are aware of the salt/sodium intake guidelines often do not follow them, as they do not want to compromise their preferred taste of food [77]. Liem et al. found that consumers added more salt to a soup when it had a “reduced-salt” label, to compensate for the perceived lack of salt in the product [85]. At primary care physician level, the barriers to implementation of dietary sodium reducing counselling are lack of time and lack of reimbursement [36]. Furthermore, the implementation of salt-reduction interventions in primary care may be further complicated by challenges in the monitoring of dietary salt intake. For example, the use of multiple 24-h urine sodium tests may not always be feasible in primary care, particularly in low resource settings [58].

Despite these challenges, health worker-led brief advice and counselling seem to be best-buy salt reduction strategies. Increasing number of healthcare providers have positive attitudes towards their role to provide guidance on salt reduction to their patients [36]. Capacity building training for health workers is required to facilitate patient counselling about sodium reduction in primary care. The World Health Organisation highlighted the importance of behaviour change communication in reducing salt intake, which would work best in the environment that promotes healthy eating [86].

## Potassium intake

The common potassium supplementation interventions in hypertensive individuals include increasing potassium intake from fruit and vegetables or using potassium supplements [19, 62].

Studies examined the effects of potassium-rich diet (e.g. DASH diet) and combined interventions that promoted potassium-rich diet, physical activity, and salt reduction on blood pressure. A study conducted in a primary care unit in Finland investigated the effect of a behavioural intervention consisting of a nurse-led counselling session to increase intake of dietary potassium, promote physical activity, and reduce salt intake on blood pressure among hypertensive patients [61]. They found no significant effects of the intervention on potassium intake and blood pressure [61].

Most of the potassium supplementation trials were conducted in controlled clinical settings rather than in primary care settings [62]. Therefore, there is a dearth of information relating to the implementation and cost of potassium supplementation interventions in primary care. Cohn et al. [87], in their review, discussed the challenges of potassium supplementation interventions in clinical practice. Before providing potassium supplementation, several factors related to the patients should be accounted for, including patients’ serum potassium levels at the time of supplementation, presence of underlying medical conditions, use of medications that alter potassium levels, dietary patterns, and ability to adhere to a dietary regimen. For example, a higher blood-pressure-lowering effect was observed among those who had a lower (< 90 mmol/day) potassium intake at baseline [62]. Furthermore, there is a U‐shaped relationship between potassium intake and blood pressure, indicating that both low and high potassium intake could result in an increased blood pressure level [88]. Patients with a comorbid condition such as congestive heart failure or chronic kidneys diseases who need to strictly maintain a given potassium level and those who use non–potassium-sparing diuretics should take precautions before commencing with potassium supplementation [89]. Recently, potassium-enriched salt substitutes were found to be effective in reducing high blood pressure [90, 91]. A study conducted in sample of 20,995 adults found that low-sodium high-potassium salt substitute not only reduced blood pressure by on average 3.34 mmHg but also reduced the risk of stroke and cardiovascular morbidity and mortality during the five-year follow-up [91]. Potassium-enriched salt substitute is a promising strategy to deal with both high dietary sodium intake and low potassium intake, while ensuring higher patient adherence, compared with low salt-high potassium diets. However, further studies are required to confirm its safety and long-term benefits in the context of hypertension.

## Physical activity

Brief Intervention and exercise referral schemes are two common physical activity promoting approaches in primary care patients*.* Brief interventions include a brief verbal advice, discussion, and encouragement with the aim to increase patient’s physical activity. Such interventions are mostly delivered by primary care practitioners such as exercise professionals, general practitioners, health coaches, health visitors, mental health professionals, midwives, pharmacists, physiotherapists, and general practice nurses [63]. A systematic review found that Brief advice on physical activity is more effective than usual care in increasing physical activity among patients [63]. The brief intervention is also cost-effective [65]. However, there is insufficient evidence regarding its effect on blood pressure, feasibility, and acceptability [92].

An exercise referral scheme, that is, a referral by a primary care or allied health professional to a physical activity specialist or service [93] was also found to be effective in increasing physical activity [64, 94]. The patients who received exercise referral increased their time in physical activity on average by 55 min more than the patients who received usual care [64]. Evidence also suggests that the compliance to physical activity recommendations following exercise referral is higher than for brief interventions [94].

However, further studies are required to confirm its cost-effectiveness. Importantly, there is a lack of evidence on the impact of exercise referral on blood pressure in hypertensive patients. It is also challenging to provide a generic recommendation for the use of exercise referral schemes in primary care, because various forms of exercise referral are being practised globally [95].

Several other types of interventions have been utilised with the aim to increase physical activity in primary care. However, they generally showed inconsistent results in increasing physical activity and lowering blood pressure. For example, three out of five studies included in the systematic review by Eden et al. [96] found a significant increase in patients’ physical activity following a clinician-led counselling intervention. In another systematic review, an intervention delivered face-to-face by health professionals was not found to be effective in increasing physical activity among patients [97]. However, for a similar intervention implemented by non-health professionals (peer health facilitators, exercise trainers) this review found a significant positive effect on physical activity [97]. Likewise, a recently published pilot study suggested that physical activity counselling for 14 weeks increases the number of steps taken per day, but has no effect on the blood pressure of hypertensive patients [98]. Significant effects on blood pressure of hypertensive patients can be expected when physical activity is combined with dietary counselling [99]. A systematic review showed that behavioural counselling on physical activity and diet reduces systolic blood pressure by on average 4.5 mmHg after 12 months and 2.3 mmHg after 12–24 months of the intervention [99].

Healthcare workers reported a lack of time and limited resources as key barriers for promoting physical activity among their patients [66]. Another study found that common facilitators and barriers for the implementation of physical activity counselling in the primary care setting are related to: practitioners’ perception of the effectiveness of physical activity in reducing hypertension; practitioners’ perception regarding patients’ interest and motivation to change their behaviour; available resources; financial incentives; conflicting priorities; and practitioners’ knowledge and confidence for prescribing physical activity [63]. The key influencing factors at the patients level are related to their motivation, the level of understanding and recall of the received advice on physical activity, fitness level, cost, lack of time, and professional, peer, family and social support [63, 67]. To address some of the barriers to promoting physical activity, Patrick et al. [100] in their review recommended healthcare delivery models that link clinical and community resources. For example, healthcare centre-based screening and advice on physical activity, followed by community support, could be a viable strategy to promote physical activity among primary care patients. Additionally, physical activity training for health workers, increased support for patients provided by providers, peers and family, and interventions tailored to the individuals’ and social needs and interests could facilitate physical activity promotion in primary care.

## Weight reduction

Behaviour change interventions and restrictive diet are commonly used with the aim to reduce weight of primary care patients. For example, a meta-analysis of 15 randomised controlled trials found an average weight reduction of 1.4 kg following a behavioural change intervention (mainly by promoting low calory diet and exercise) for weight loss [68]. The behavioural change interventions are usually delivered by primary care physicians and nurses, psychologists, health educators, and nutritionists [68]. They encompass self-monitoring of diet and exercise behaviour, followed by behavioural goal setting and barrier identification or problem-solving [68]. A recent study found that a healthcare provider-led weight reduction discussion was associated with 5% greater weight reduction among overweight and obese patients, compared with patients who did not participate in such discussion [101]. Likewise, a brief counselling provided by a primary care physician resulted in an average weight loss of around 2.5 kg at 6 to 12 months follow-ups [102]. Daumit et al. [70] further suggested that a primary care-based behavioural change intervention (reducing calorie intake, increasing physical activity, and self-monitoring) would be more cost-effective if it is administered remotely (e.g. by telephone) than in person.

Furthermore, a study compared the costs of doctor’s referral to a commercially provided restrictive diet and a nurse-led behaviour change support for reducing body weight in obese patients [71]. The former was found to be more cost-effective for the routine treatment of obesity in healthcare settings [71]. Evidence also indicates that low-energy diets are more effective for weight reduction in the short term, compared with behavioural therapy [69, 71, 103]. However, their use is recommended only when a rapid weight reduction is required, and they should only be provided by trained professionals and alongside regular medical monitoring to prevent adverse events [69]. This may reduce their feasibility in the primary care setting. It is also important to note that a large regain of lost weight (> 40% for low-energy diets and > 60% for very-low-energy diets) is expected within 1–5 years [104]. Although restrictive diets are associated with a reduction in blood pressure [105–107], very little is known about their long-term impact on other aspects of health of people with hypertension [105].

A lack of self-motivation, a lack of self-control, inability to afford healthy foods and exercise equipment, inability to resist the temptation for unhealthy foods, competing priorities, and comorbidities are some of the impediments for weight loss [72, 73]. By contrast, higher self-motivation, incentives, rewards, and peer, professional and social support could facilitate weight loss in the long term [72]. Barriers at the level of primary care workers include: the reluctance to discuss weight management with patients; insufficient confidence, knowledge and skill to manage weight; lack of clear guidelines for weight reduction; limited resources; lack of time; and physicians’ pessimism about patients’ weight loss success [73, 74].

Primary care-based weight-reduction interventions consisting of both reduced energy intake and increased physical activity are more effective than interventions with any of these components individually [108]. Enabling access to dieticians and exercise professionals, and addressing barriers at the levels of providers and patients should be a priority in future interventions.

## Heart-healthy diets

Heart-healthy diets typically include the diets with high intake of fruits and vegetables, low fat intake, consumption of whole grains, and low sodium intake. The two most commonly used dietary approaches for hypertension control are DASH and Mediterranean diet [28, 109]. They are mostly delivered by dietary education through face-to-face counselling [60] or via telephone or email [59]. They are usually delivered by primary care physicians [110], nurses, dieticians [59], nutritionists [60], and other health workers [110]. The dietary interventions are often combined with exercise, weight loss, and salt reduction interventions to achieve better results [111, 112]. The effectiveness of DASH diet for reducing blood pressure in primary care is limited. Recent studies from Brazil [60] and Hong Kong [113] did not find a significant effect of dietary counselling on blood pressure in primary care patients.

Furthermore, while implementing dietary intervention in a primary care setting it may be challenging to provide heart-healthy meals to patients and adequate counselling [55]. In addition, it is found that adherence to dietary recommendations is relatively low among patients [114]. Some of the reasons for non-adherence to DASH diet as perceived by the healthcare providers are low patient motivation, lack of provider time, and lack of educational resources for patients [75]. Primary care physicians from Canada stated that the lack of time, difficulty in assessing patient’s dietary pattern, patient’s non-adherence to dietary advice, and inconsistent dietary guidelines are the major barriers to implementation of DASH diet intervention in primary care [76]. From hypertensive patient’s perspective, the major barriers to following a recommended diets are social and environmental factors such as eating outside the home or eating food cooked by others, and lack of food choice in social gathering; lack of family support; lack of taste in diet; and cost of diet [115].

The physicians from Canada also stated that the use of electronic medical record tools that support dietary screening or counselling, access to dietitian support, and nutrition education as part of medical training would help them provide dietary advice to patients [76].

## Other promising non-pharmacological interventions

Emerging evidence suggests that other non-pharmacological interventions such as yoga, stress reduction, and healthy drinks could be beneficial for reducing blood pressure [27, 30, 34]. For example, a recent meta-analysis of 49 clinical trials found that engaging in three sessions per week of yoga (including breathing techniques and meditation/mental relaxation) is associated with an average reduction in systolic blood pressure of 5 mmHg [34]. A systematic review suggested that a mindfulness-based stress reduction program is a promising behavioural therapy for reducing blood pressure in people with hypertension [116]. Studies also suggested that moderate consumption of coffee and green tea could be beneficial for reducing blood pressure [117, 118].

However, evidence on the effectiveness of these interventions in the primary care setting is limited. Only a few studies investigated the effects of yoga interventions delivered in the primary care setting on blood pressure of hypertensive patients while utilising a primary care physician to provide yoga instruction. For example, Wolf et al. conducted two such studies in Sweden [119, 120]. Their first study found an average reduction in diastolic blood pressure of around 4 mmHg, following a 12 weeks intervention. However, in their subsequent study, they did not find a statistically significant effect [120]. Dhungana et al. found that a health worker-led 3-month yoga intervention significantly reduced systolic blood pressure in hypertensive patients on average by 7.66 mmHg [121].

Regarding stress reduction, a private clinic-based study found that participation in eight 2.5-h mindfulness-based stress reduction sessions was associated with a reduction of around 12 mmHg of systolic blood pressure [122]. Although there is a dearth of evidence on the effect of stress reduction interventions on blood pressure in primary care settings, a number of studies indicated that mindfulness-based interventions are promising for improving mental health and are feasible to be implemented in primary care settings [123, 124]. Studies have also explored the potential role of green and black tea for blood pressure reduction [118]. However, no studies have investigated their applicability by physicians and health care providers for hypertension management in primary care.

## Conclusion

Non-pharmacological interventions for the treatment of hypertension in primary care with proven effectiveness include alcohol reduction. Intervention for sodium intake reduction, physical activity, and weight reduction is effective for blood pressure reduction, but it requires more pragmatic, clinically feasible, and logistically simple method in outpatients setting. Evidence on the effectiveness of potassium intake and heart-healthy diets is limited and inconsistent.

Given that studies have estimated only the overall cost-effectiveness of implementing non-pharmacological interventions (e.g. reduced alcohol intake, increased physical activity, weight loss), there is a lack of specific information on the cost-effectiveness of these interventions in the treatment of hypertension.

The most common barriers to deliver such interventions related to healthcare providers include a lack of time, knowledge, self-confidence, resources, clear guidelines, and financial incentives. The most common barriers related to patients include a lack of motivation and educational resources.

Less evidence is available on facilitators of implementing non-pharmacological interventions in primary care. Besides, facilitators differed by different types of interventions.

Based on the current evidence, healthcare providers should consider implementing alcohol reduction, sodium intake reduction, physical activity, and weight reduction interventions for blood pressure reduction in the primary care setting.

Future studies should provide further evidence on the effectiveness of weight control, potassium intake, and heart-healthy diets. More research is also needed on cost-effectiveness and facilitators of all types of effective non-pharmacological interventions for the treatment of hypertension in primary care.

## Data Availability

All data generated or analysed during this study are included in this article.
